# Dexamethasone induces sleep disturbance in a dose-dependent manner in mice

**DOI:** 10.1371/journal.pone.0296028

**Published:** 2023-12-20

**Authors:** Tomonobu Kato, Gento Okawa, Kenji F. Tanaka, Yasue Mitsukura

**Affiliations:** 1 Faculty of Science and Technology of Keio University, Yokohama, Kanagawa, Japan; 2 Division of Brain Sciences, Institute for Advanced Medical Research, Keio University School of Medicine, Shinjuku-ku, Tokyo, Japan; University of Nebraska Medical Center College of Medicine, UNITED STATES

## Abstract

Synthetic corticosteroids, the most well-known anti-inflammatory drugs globally, are effective against inflammatory diseases despite their adverse effects that decrease a patient’s quality of life (QOL). One of these effects is sleep disturbance, which causes other health issues and further diminishes the QOL. However, the acute effects of steroid drugs on sleep-wake issues are not fully understood and must be clarified in detail using experimental animals. Therefore, this study examines the dose-dependent effect of dexamethasone (DXM), one of the strongest steroid drugs, on the sleep-wake architecture of mice. We conducted acute DXM administration at multiple doses and 24-hour EEG/EMG recordings. Our results revealed that DXM increased the time spent in arousal and decreased that of NREM sleep, even at very low doses. These results imply that steroid-induced sleep disturbance must be addressed at any dosage.

## Introduction

Synthetic steroids are widely used anti-inflammatory drugs for treating various diseases, such as dermatologic, ophthalmologic, rheumatologic, pulmonary, hematologic, and gastrointestinal (GI) disorders. However, they can cause side effects such as osteoporosis and fractures, adrenal suppression, hyperglycemia and diabetes, cardiovascular disease and dyslipidemia, dermatological problems, GI events, psychiatric disturbances, and immunosuppression [1]. Psychiatric and cognitive disturbances contain a wide range of conditions, including memory impairment, agitation, anxiety, fear, hypomania, sleep disturbance, irritability, lethargy, mood lability, and even psychosis. These are common side effects of systemic corticosteroid therapy―severe reactions occur in nearly 6% of patients and mild to moderate reactions occur in approximately 28% of patients [2,3]. Psychiatric disturbances can occur early in the therapeutic course; for example, Hall, et al. [4] reported that 86% of patients suffer a psychiatric adverse event within the first week of treatment.

Dexamethasone (DXM) is a synthetic corticosteroid developed as a drug with relatively few side effects and strong anti-inflammatory properties. However, one of its most frequently reported adverse effects in patients is sleep disturbance [5]. Sleep disturbance causes other health issues such as cardiovascular disease [6]; therefore, it is important to address these adverse effects in daily clinical practice. Steroid pulse therapy with DXM causes more complaints of sleep disturbance than in the case of prednisolone [7], one of the long-established synthetic steroids [8]. Moser et al. [9] showed that 3 mg of DXM administration every eight hours (total 48 h) caused an increase in the REM sleep latency and percentage of time spent in arousal, as well as a decrease in REM sleep and the number of REM sleep periods in healthy humans. Demisch et al. [10] showed that acute administration of DXM (1 mg) changed the production of nocturnal melatonin (sleep-wake cycle regulator), indicating that steroid-induced sleep disturbance requires only one dose of DXM induction. In the animal model, an administration of 0.5 mg/kg DXM once daily for 21 consecutive days causes an increase in the power of slow EEG frequencies during wake state and decreases the time spent in REM sleep [11].

However, although steroid-induced sleep disturbance occurs immediately after acute DXM administration, the resulting changes in sleep architecture have not been adequately evaluated. Additionally, although the half-life of dexamethasone (DXM) is 190 min, it remains in the blood as a glucocorticoid for 12 h, and its anti-inflammatory effects last for 36–54 h (Peter et al., 2017). Chronic DXM administration can potentiate a high incidence rate of side effects, as these effects can overlap. Therefore, we evaluated the intensity and duration of sleep disturbance caused by acute DXM administration to reveal the true potential of DXM for causing sleep disturbance.

In this study, we performed 24-hour electroencephalography/electromyography (EEG/EMG) recordings following acute DXM administration at multiple doses in mice and revealed that one-time DXM administration increases the time spent in the wake state in a dose-dependent manner and occurs even in very low dose ranges.

## Materials and methods

### Ethics statement

This study was carried out in strict accordance with the recommendations in the Guide for the Care and Use of Laboratory Animals of the National Institutes of Health. The protocol was approved by the Keio University Institutional Animal Care and Use Committee (approval number A2022-315). All surgery was performed under a mixture of ketamine and xylazine anesthesia, and all efforts were made to minimize suffering.

### Animals

Experiments were conducted using 8 to 14 month old male and female wild-type (129 SvEvTac) mice. All mice were kept in a 12:12 h light/dark cycle (light on at 08:00 a.m.; luminous flux 120 lm), and polysomnographic recordings were performed during the light phase (start time at 08:00 a.m. [Zeitgeber Time (ZT) 0]). All the mice were monitored daily by trained members of the research team for food and water consumption and overall health status, with no adverse conditions or health outcomes noted. All the mice were sacrificed by cervical dislocation after the recordings were completed. The animal room was maintained at 24°C and 50% humidity. All the animals were provided with a laboratory diet (CL-2, CLEA Japan) and tap water.

### Surgical procedure

Surgeries were performed using a stereotaxic apparatus (SM-6M-HT, Narishige, Tokyo, Japan). Mice were anesthetized via intraperitoneal administration using a mixture of ketamine and xylazine (100 and 10 mg/kg, respectively). The mice received permanent EEG and EMG electrode implants for polysomnography. Three pits were drilled into the skull using a carbide cutter (drill size diameter 0.8 mm). Each implant had a 1.0 mm diameter stainless steel screw that served as an EEG electrode; one implant was placed on the right frontal cortical area (AP, +1.0 mm; ML, +1.5 mm) as a reference electrode and the other was positioned over the right parietal area (AP, +1 mm anterior to the lambda; ML, +1.5 mm) as a signal electrode. Another electrode was placed over the right cerebellar cortex (AP, −1.0 mm posterior to lambda; ML, +1.5 mm) as a ground electrode. Two silver wires (catalog #AS633, Cooner wire, Chatsworth, CA, USA) were placed bilaterally into the trapezius muscles and served as EMG electrodes. Finally, the electrode assembly was anchored and fixed to the skull using SuperBond (Sun Medical, Shiga, Japan).

### EEG/EMG recordings

The EEG/EMG signals were amplified (gain ×1000) and filtered (EEG:1–300 Hz, EMG:10–300 Hz) using a DC/AC differential amplifier (AM-3000, AM systems, Sequim, WA, USA). The input was then received via an input module (NI-9215, National Instruments, Austin, TX, USA), digitized at a sampling rate of 1000 Hz using a data acquisition module (cDAQ-9171, National Instruments, Austin, TX, USA), and recorded using a custom-made LabVIEW program (National Instruments, Austin, TX, USA). We habituated the 24-hour EEG/EMG recordings more than three times; when REM sleep (see vigilance state assessment) was consistently observed, the experiment was initiated.

### Dexamethasone administration

Following habituation, we injected saline intraperitoneally at ZT0 and conducted 24-hour EEG/EMG recordings as a control. After 3 d, we injected dexamethasone (150 mg/kg or 15 mg/kg or 1.5 mg/kg or 0.15 mg/kg) intraperitoneally at ZT0 into the same mice and conducted 24-hour EEG/EMG recordings.

### Mouse vigilance state assessment

The EEG/EMG signals were analyzed using MATLAB (MathWorks, Cambridge, MA, USA). Power-spectral EEG data were obtained using the multi-spectrogram method. A power spectral profile was used for the analysis. We detected each sleep-wake state scored offline by visually characterizing 10 s epochs, as previously described [12,13]. The wake state was characterized by a low-amplitude fast EEG and a high-amplitude variable EMG. Non-REM (NREM) sleep was characterized by high-amplitude delta (1–4 Hz) frequency EEG and low-amplitude tonus EMG. REM sleep was staged using theta (4–9 Hz) dominant EEG and EMG atonia [14].

### Data processing

All animals and trials were randomly assigned to experimental conditions. The experimenters were not blinded to the experimental conditions during data collection and analysis, and EMG signals were analyzed using custom-made programs in MATLAB. The EEG signals were bandpass filtered (finite impulse response [FIR], Kaiser window). The EEG frequency bands were as follows: delta: 1–4 Hz, theta: 4–9 Hz, sigma: 9–12 Hz, beta: 12–30 Hz, and gamma: 30–50 Hz [15,16]. We obtained the power of each EEG frequency band using the square of the amplitude. Additionally, we used Hampel and 50 Hz notch filters for EMG signals to remove both chatter and hum noises.

### Statistical analysis

All experimental data were analyzed using the following parametric statistics: paired *t*-test, one-way ANOVA, followed by the Tukey-Kramer *post-hoc* test. To compare sleep structures, paired *t*-tests were used because control data and after DXM administration data were paired. When comparing sleep structure changes across DXM doses, a one-way ANOVA was conducted, followed by Tukey-Kramer *post-hoc* corrections for multiple comparisons.

## Results

### Dexamethasone increased wakefulness and decreased NREM sleep after a single injection in a dose-dependent manner

**Fig 1:**
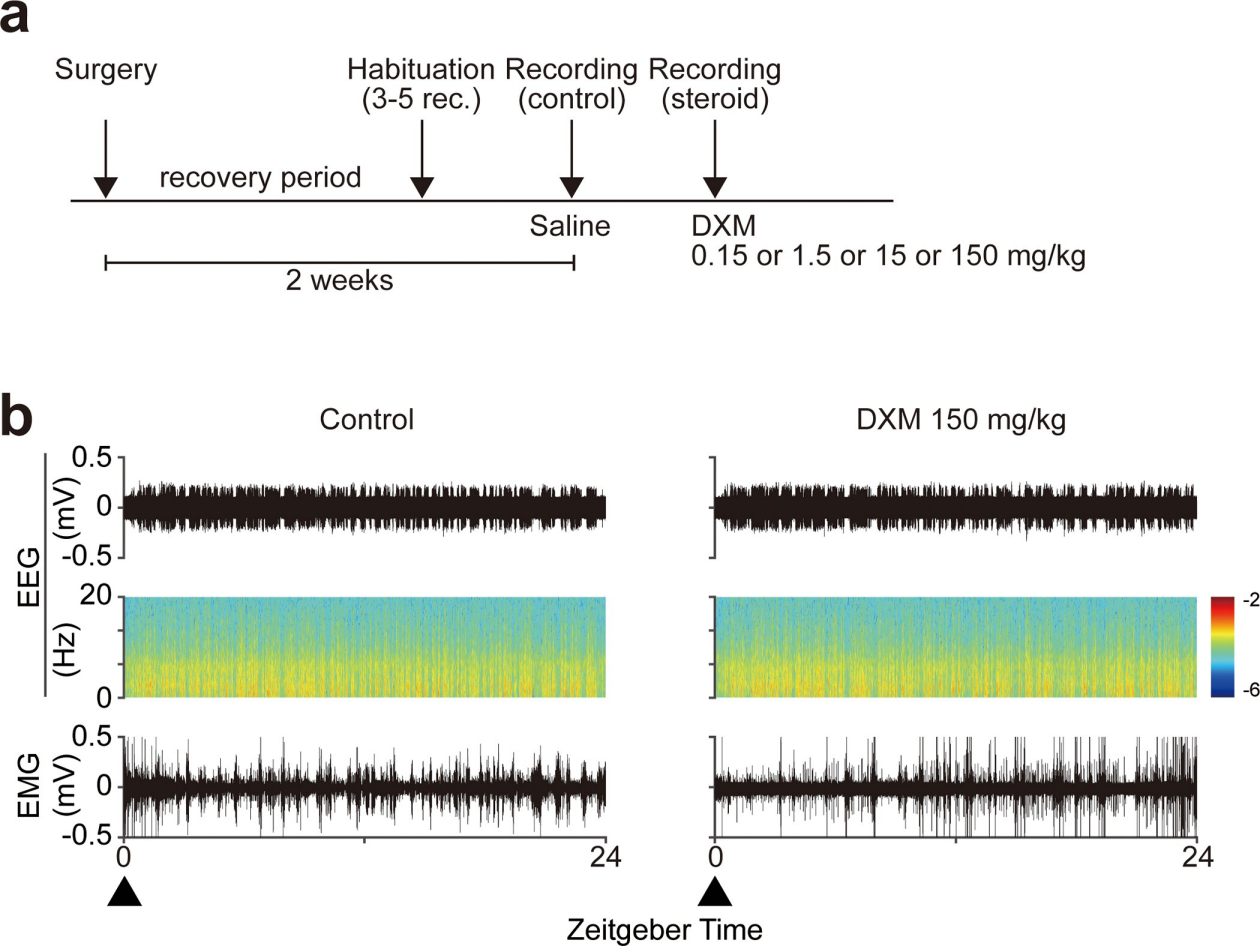
Experimental protocol and representative data. (a) Experimental flow. After surgery and a 1 week recovery period, we recorded the mice for habituation. After that, saline was injected at ZT0 and 24-hour EEG/EMG recording was conducted as a control. Two days later, we injected DXM (0.15 mg/kg or 1.5 mg/kg or 15 mg/kg, 150 mg/kg) at ZT0 and recorded 24-hour EEG/EMG results. (b) Representative data. Left, control; right, DXM injection. The upper row shows the EEG signal, the middle row shows the relative EEG power spectrogram, and the lower row shows EMG signal. Right, following 150mg/kg of DXM administration. The upper row shows the EEG signal, the middle row shows the relative EEG power spectrogram, and the lower row shows the EMG signal. The black triangle shows the DXM injection time.

**Fig 2:**
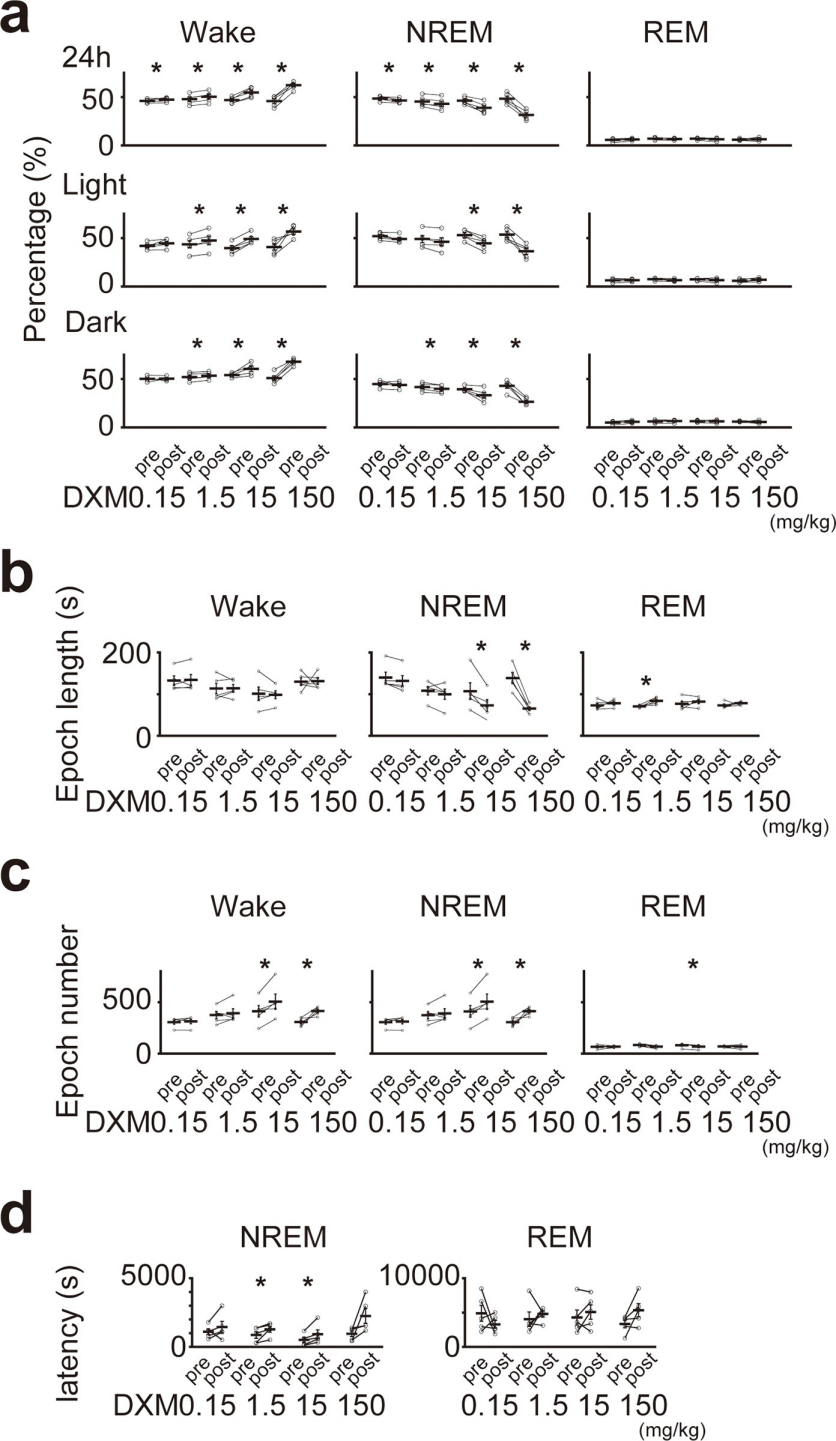
DXM administration increases time spent in the wake state and decreases that in NREM sleep. (a) The percentage of each sleep state following each dose of DXM administration. The upper row shows the daily total, the second row shows the light phase, and the third row shows the dark phase. The left column shows the wake state, the middle column shows the NREM sleep state, and the right column shows the REM sleep state. The results of the statistical test are shown in Table 1 (n = 5 mice in each dose of DXM, total 20 mice; paired *t*-test). (b) The duration of each sleep-wake state before and after DXM administration. The left column shows the wake state, the middle column shows the NREM sleep state, and the right column shows the REM sleep state. The results of the statistical test are shown in Table 2 (n = 5 mice in each dose of DXM, total 20 mice; paired *t*-test). (c) Total number of sleep-wake state occurrences observed before and after DXM administration. The left column shows the wake state, the middle column shows the NREM sleep state, and the right column shows the REM sleep state. The results of the statistical test are shown in Table 3 (n = 5 mice in each dose of DXM, total 20 mice; paired *t*-test). (d) NREM (left panel) and REM (right panel) latency before and after DXM administration. The results of the statistical test are shown in Table 4 (n = 5 mice in each dose of DXM, total 20 mice; Paired *t*-test). * < 0.05. W indicates the wake state, NR indicates NREM sleep, R indicates REM sleep. ‘pre’ means saline administration (control experiment), ‘post’ means DXM administration. The error bar shows S.E.M.

To examine the effects of acute dexamethasone (DXM) administration on sleep architecture, we compared the results of 24-hour polysomnography immediately after DXM administration with those after the same dose of saline (Fig 1A and 1B). Furthermore, we compared four different doses of DXM to examine dose-dependent effects (Fig 1A). Fig 2A shows the changes in the sleep architecture between the control and following DXM administration. In total, the time spent in the wake state was significantly increased from 45.8 ± 0.02 to 62.3 ± 0.02, 46.9 ± 0.01 to 54.8 ± 0.02, 47.7 ± 0.02 to 50.3 ± 0.02, and 46.0 ± 0.01 to 47.4 ± 0.01%, respectively (150, 15, 1.5, 0.15 mg/kg, respectively; mean ± SEM), whereas that in the NREM state significantly decreased from 48.3 ± 0.03 to 31.4 ± 0.02, 46.2 ± 0.02 to 38.8 ± 0.03, 45.3 ± 0.02 to 43.1 ± 0.03, and 48.4 ± 0.01 to 46.5 ± 0.01%, respectively (150, 15, 1.5, 0.15 mg/kg respectively; mean ± SEM). In the light phase, the time spent in the wake state significantly increased after 150, 15 and 1.5 mg/kg of DXM administration (40.8 ± 0.03 to 56.7 ± 0.03, 39.7 ± 0.02 to 49.0 ± 0.02, 43.5 ± 0.04 to 47.4 ± 0.04% respectively; mean ± SEM), whereas that in the NREM sleep decreased after 150 and 15 mg/kg of DXM administration (53.4 ± 0.03 to 36.4 ± 0.03, 53.0 ± 0.02 to 44.5 ± 0.03% respectively; mean ± SEM). In the dark phase, the time spent in the wake state significantly increased and that in NREM sleep significantly decreased after 150, 15 and 1.5 mg/kg of DXM administration ([wake] 51.0 ± 0.02 to 67.9 ± 0.02, 54.1 ± 0.01 to 60.6 ± 0.03, 52.0 ± 0.02 to 53.5 ± 0.02; [NREM] 42.9 ± 0.03 to 26.5 ± 0.02, 39.5 ± 0.01 to 33.2 ± 0.03, 41.8 ± 0.02 to 39.9 ± 0.02% respectively; mean ± SEM). Additionally, the time spent in the NREM sleep after 1.5 mg/kg of DXM administration in the light phase decreased, and the time spent in the wake state and in NREM sleep after 0.15 mg/kg of DXM administration in both the light and the dark phases increased and decreased, respectively ([1.5 mg/kg, NREM] 48.9 ± 0.04 to 46.1 ± 0.04; [0.15 mg/kg, wake state], 41.9 ± 0.02 to 44.5 ± 0.02 [light phase], 50.2 ± 0.01 to 50.3 ± 0.01 [dark phase]; [0.15 mg/kg, NREM] 51.9 ± 0.02 to 49.0 ± 0.02 [light phase], 44.8 ± 0.01 to 43.9 ± 0.01 [dark phase] %; mean ± SEM). Subsequently, we compared the duration of each sleep-wake epoch and observed that the duration for NREM sleep decreased after 150 mg/kg and 15 mg/kg of DXM administration (138.6 ± 12.9 to 65.6 ± 4.4, 107.4 ± 20.0 to 73.2 ± 13.9 sec, respectively; mean ± SEM; Fig 2B). Furthermore, the number of wake and NREM sleep epochs increased after 150 mg/kg and 15 mg/kg of DXM administration ([wake] 307.0 ± 18.3 to 414.2 ± 18.7, 411.4 ± 55.6 to 506.2 ± 72.5; [NREM] 306.6 ± 18.4 to 413.2 ± 18.5, 410.8 ± 55.8 to 505.6 ± 72.5 epochs, respectively; mean ± SEM; Fig 2C). These results indicated a fragmentation of NREM sleep following 150 mg/kg and 15 mg/kg of DXM administration. Furthermore, NREM sleep latency was prolonged, and was particularly and significantly changed following 15 mg/kg and 1.5 mg/kg of DXM administration (960.8 ± 204.9 to 2253.2 ± 533.9, 512.8 ± 187.9 to 925.8 ± 317.3, 866.2 ± 242.8 to 4040.2 ± 1053.2, and 1106.2 ± 206.8 to 1442.4 ± 418.5 sec for 150, 15, 1.5 and 0.15 mg/kg, respectively; mean ± SEM). REM sleep latency also increased, except that for 0.15 mg/kg. However, it did not significantly change in all dosages (3347.2 ± 567.9 to 5346.4 ± 964.8, 4281.6 ± 1100.6 to 5082.2 ± 1042.7, 4040.2 ± 1053.2 to 4802.4 ± 454.4, and 4894.2 ± 1167.2 to 3284.6 ± 579.6 sec for 150, 15, 1.5 and 0.15 mg/kg, respectively; mean ± SEM; Fig 2D).

**Table 1:** Results of paired t-test for the percentage of each sleep state after each dose of DXM administration for the data in Fig 2A.

				*P* Value					
	Wake		*	NREM			REM		
DXM (mg/kg)	24h	light	dark	24h	light	dark	24h	light	dark
150	1.7×10^−3^*	0.02*	7.6×10^−4^*	3.1×10^−3^*	0.02*	2.1×10^−3^*	0.55	0.11	0.62
15	2.1×10^−3^*	6.0×10^−4^*	0.03*	4.4×10^−3^*	2.4×10^−3^*	0.03*	0.19	0.12	0.76
1.5	2.0×10^−4^*	5.6×10^−3^*	4.1×10^−3^*	0.01*	0.06	0.01*	0.62	0.08	0.37
0.15	0.04*	0.11	0.92	0.04*	0.11	0.46	0.23	0.37	0.2

*p < 0.05

**Table 2:** Results of paired t-test for total number of sleep-wake state occurrences before and after DXM administration for the data in Fig 2B.

		*P* Value	
DXM (mg/kg)	Wake	NREM	REM
150	0.95	1.3×10^−3^*	0.35
15	0.77	0.02*	0.40
1.5	0.96	0.26	0.04*
0.15	0.76	0.05	0.50

*p < 0.05.

**Table 3:** Results of paired t-test for the duration of each sleep-wake state before and after DXM administration for the data in Fig 2C.

		*P* Value	
DXM (mg/kg)	Wake	NREM	REM
150	0.02*	0.02*	0.94
15	0.04*	0.03*	0.01*
1.5	0.53	0.52	0.14
0.15	0.49	0.53	0.85

*p < 0.05.

**Table 4:** Results of paired t-test for NREM and REM latency before and after DXM administration for the data in Fig 2D.

	*P* Value	
DXM (mg/kg)	NREM	REM
150	0.09	0.10
15	4.9×10^−2^*	0.39
1.5	4.9×10^−2^*	0.55
0.15	0.33	0.36

*p < 0.05.

**Fig 3:**
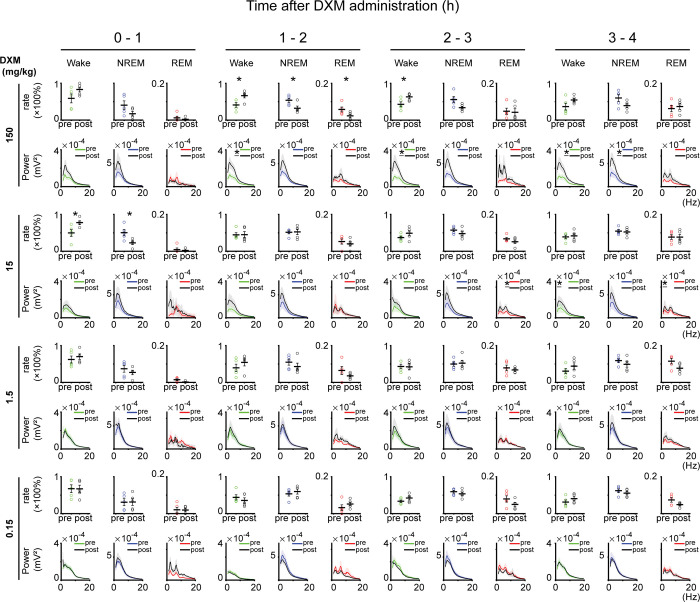
Change in sleep structure per hour for the first four hours after DXM administration. The percentages of wake and sleep states per hour for the first four hours of DXM administration and EEG spectrogram of the same times. The figure is arranged from left to right along the time axis, with higher steroid doses at the top and lower doses at the bottom. In each area, the upper row shows the percentage of wake, NREM, and REM sleep states, and the lower row shows EEG spectrograms of the wake, NREM, REM sleep states. In the column for sleep/wake state times, the left bar shows the control, and the right bar shows DXM administration. In the column for EEG spectrum, the colored line (green or blue or red) shows the control, and the black line shows DXM administration. ‘pre’ means saline administration (control experiment), ‘post’ means DXM administration. The results of statistical tests are shown in Tables 5 and 6.

Next, we evaluated the time spent in each sleep/wake state per hour after the first four hours of DXM administration to determine whether the increase in wakefulness occurred immediately following DXM administration and the temporal progression of such changes (Fig 3, Table 3). For 150 mg/kg of DXM, there was a significant increase at 1–2 h after DXM administration with 41.3 ± 0.06 vs. 66.5 ± 0.06% (*p* = 0.02, paired *t*-test) and at 2–3 h with 43.0 ± 0.07 vs. 63.2 ± 0.04% (*p* = 0.04, paired *t*-test); a similar increasing trend was observed at 0–1 h with 58.9 ± 12.1 vs. 83.0 ± 0.07%, and at 3–4 h with 36.5 ± 0.09 vs. 54.5 ± 0.05%. For 15 mg/kg of DXM, there was a significant increase in the percentages of wakefulness at 0–1 h after DXM administration with 49.7 ± 0.09 vs. 77.8 ± 0.05% (*p* = 0.04, paired *t*-test). Additionally, the same increasing trend was observed, but there were no significant differences at 2–3 h with 37.0 ± 0.04 vs. 48.8 ± 0.07%, and at 3–4 h with 38.9 ± 0.05 vs. 41.8 ± 0.06%. For 1.5 mg/kg of DXM, there was an increasing trend in the percentages of wakefulness, with 62.2 ± 0.09 vs. 70.0 ± 0.07% at 0–1 h after DXM administration, 39.7 ± 0.10 vs. 54.4 ± 0.10% at 1–2 h, and 30.1 ± 0.07 vs. 44.2 ± 0.09% at 3–4 h. The only exception was at 2–3 h, where no such trend was observed with 43.3 ± 0.06 vs. 42.3 ± 0.08%. For 0.15 mg/kg of DXM administration, there was no significant difference in the percentages of wakefulness during the first 4 h after administration. However, at 2–3 h after DXM administration, an increasing trend was observed with 33.5 ± 0.03 vs. 42.3 ± 0.05% (control vs. DXM administration, respectively, mean ± SEM; the same convention is used below), and at 3–4 h, the same trend was observed with 31.3 ± 0.06 vs. 40.1 ± 0.05%. There was individual variation in the period at which arousal time began to increase after DXM administration, thereby ensuring that no consistent trend was observed. However, with higher doses (150 and 15 mg/kg) of DXM administration, a larger increase in the duration of wakefulness was observed compared to lower doses (1.5 and 0.15 mg/kg).

**Table 5:** Results of paired t-test for percentages of sleep/wake times between before and after DXM administration for the data in Fig 3.

			*P* Value	
DXM (mg/kg)	Time	Wake	NREM	REM
150	0-1h	0.14	0.13	0.48
	1-2h	0.02*	0.02*	0.02*
	2-3h	0.04*	0.06	0.77
	3-4h	0.23	0.18	0.67
15	0-1h	0.04*	0.03*	0.68
	1-2h	0.97	0.88	0.23
	2-3h	0.15	0.2	0.19
	3-4h	0.52	0.63	0.99
1.5	0-1h	0.54	0.28	0.12
	1-2h	0.09	0.17	0.17
	2-3h	0.90	0.74	0.50
	3-4h	0.08	0.11	0.06
0.15	0-1h	0.98	0.97	0.93
	1-2h	0.27	0.40	0.24
	2-3h	0.16	0.43	0.15
	3-4h	0.36	0.46	0.13

*p < 0.05.

**Table 6:** Results of paired t-test for EEG delta and theta power between before and after DXM administration for the data in Fig 3.

				*P* Value			
		Wake		NREM		REM	
DXM (mg/kg)	Time	delta	theta	delta	theta	delta	theta
150	0-1h	0.09	0.09	**	**	0.60	0.56
	1-2h	0.07	0.03*	0.09	0.06	**	**
	2-3h	0.06	0.04*	0.06	0.05	0.09	0.05
	3-4h	0.07	4.9×10^−2^*	0.08	4.9×10^−2^*	0.14	0.15
15	0-1h	0.07	0.05	0.09	0.13	**	**
	1-2h	0.06	0.10	0.09	0.21	0.76	0.50
	2-3h	0.08	0.22	0.06	0.09	0.11	0.04*
	3-4h	0.04*	0.09	0.09	0.15	0.03*	0.10
1.5	0-1h	0.29	0.63	0.36	0.34	0.43	0.47
	1-2h	0.21	0.21	0.48	0.42	**	**
	2-3h	0.21	0.27	0.29	0.39	0.39	0.53
	3-4h	0.28	0.56	0.21	0.29	0.15	0.13
0.15	0-1h	0.40	0.56	0.48	0.26	**	**
	1-2h	0.62	0.39	0.37	0.32	**	**
	2-3h	0.61	0.39	0.40	0.59	0.27	0.29
	3-4h	0.93	0.52	0.59	0.58	0.40	0.61

*p < 0.05.

**The paired t-test is not applicable because some data is missing (Sleep is absent).

**Fig 4:**
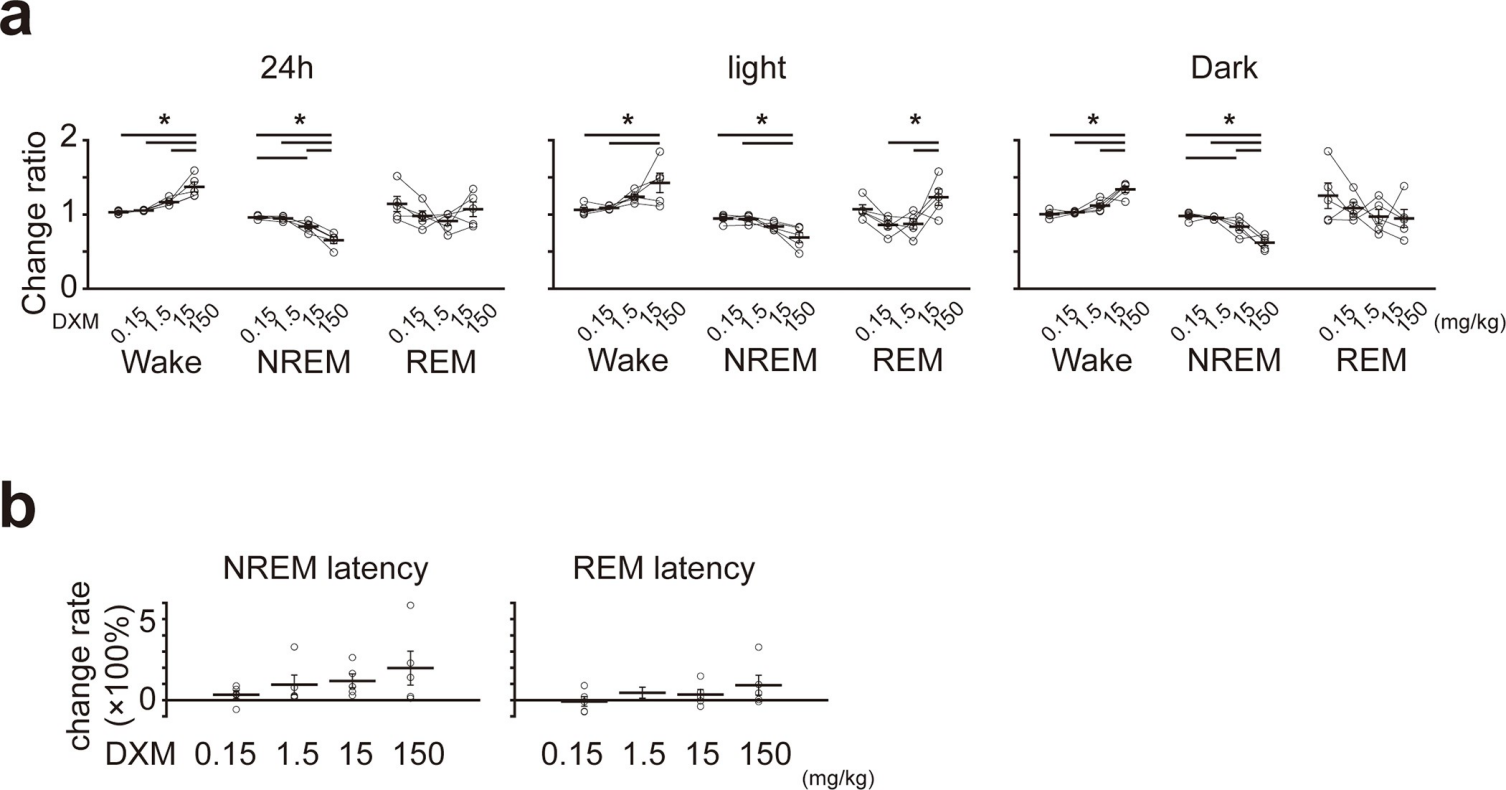
Change in sleep structure after DXM administration showing a dose-dependent dynamic. (a) The ratio of each amount of sleep-wake state before and after DXM administration in each dose. The left column shows the daily total, the middle column shows the light phase, the right column shows the dark phase. In each figure, the left column shows the time spent in the wake state, the middle column shows the time spent in NREM sleep, and the right column shows the time spent in REM sleep. The results of the statistical tests are shown in Table 7 (n = 5 mice in each dose of DXM; total 20 mice. One-way ANOVA followed by the Tukey-Kramer *post hoc* test). (b) Change in the latency of NREM (left panel) and REM (right panel) after DXM administration between each dose. The results of the statistical tests are shown in Table 8 (n = 5 mice in each dose of DXM; total 20 mice. One-way ANOVA followed by Tukey-Kramer *post hoc* test). * < 0.05. The error bar shows S.E.M.

Furthermore, to evaluate dose dependency, we examined the ratio of sleep-wake state amount before and after DXM administration in each dose (Fig 4A). For 150 mg/kg of DXM, the time spent in the wake state increased 1.37 ± 0.07 (mean ± SEM) times compared to the control. In contrast, for 15 mg/kg, it was 1.17 ± 0.02; for 1.5 mg/kg, it was 1.05 ± 2.7 × 10^−3^, and for 0.15 mg/kg, it was 1.03 ± 0.01 times (mean ± SEM), showing a dose-dependent relationship in the degree of increase. Significant differences were observed between 150 mg/kg and 0.15 mg/kg, 150 mg/kg and 1.5 mg/kg, and 150 mg/kg and 0.15 mg/kg of DXM. Similar trends were observed when analyzing the light and dark phases separately. However, the time spent in the NREM sleep decreased in the same manner, at 0.65 ± 0.04, 0.84 ± 0.03, 0.95 ± 0.01, and 96.1 ± 0.01 times for 150, 15, 1.5, and 0.15 mg/kg, respectively. Significant differences were observed between 150 mg/kg and 0.15 mg/kg, 150 mg/kg and 1.5 mg/kg, 150 mg/kg and 0.15 mg/kg, and 15 mg/kg and 0.15 mg/kg of DXM. Based on these results, DXM-induced sleep disturbance was dose-dependent in mice. However, the change rate of NREM and REM sleep latency did not show significant differences between each DXM amount (Fig 4B).

**Table 7:** Results of one-way ANOVA followed by the Tukey-Kramer *post hoc* test for the ratio of each amount of sleep-wake state following DXM administration for the data in Fig 4A.

					*P* Value					
		Wake			NREM			REM		
DXM (mg/kg)		24h	light	dark	24h	light	dark	24h	light	dark
150 vs	15	3.9×10^−3^*	0.27	5.6×10^−4^*	1.6×10^−3^*	0.08	3.0×10^−3^*	0.55	0.02*	1
	1.5	4.4×10^−5^*	0.02*	1.1×10^−5^*	1.0×10^−5^*	2.6×10^−3^*	3.1×10^−5^*	0.87	0.02*	0.85
	0.15	1.9×10^−5^*	0.01*	4.3×10^−6^*	5.7×10^−6^*	2.0×10^−3^*	1.3×10^−5^*	0.93	0.46	0.33
15 vs	1.5	0.14	0.45	0.19	0.07	0.34	0.13	0.94	0.99	0.91
	0.15	0.06	0.32	0.07	0.04*	0.28	4.9×10^−2^*	0.25	0.29	0.39
1.5 vs	0.15	0.96	0.99	0.94	0.99	0.99	0.95	0.53	0.24	0.77

*p < 0.05.

**Table 8:** Results of one-way ANOVA followed by the Tukey-Kramer *post hoc* test for the rate of change in the latency of NREM and REM after DXM administration for the data in Fig 4B.

		*P* Value	
DXM (mg/kg)		NREM	REM
150 vs	15	0.91	0.79
	1.5	0.79	0.89
	0.15	0.32	0.35
15 vs	1.5	0.99	1
	0.15	0.69	0.85
1.5 vs	0.15	0.83	0.76

*p < 0.05.

### EEG power in each frequency band showed minimal changes and almost no significant difference after DXM administration

**Fig 5:**
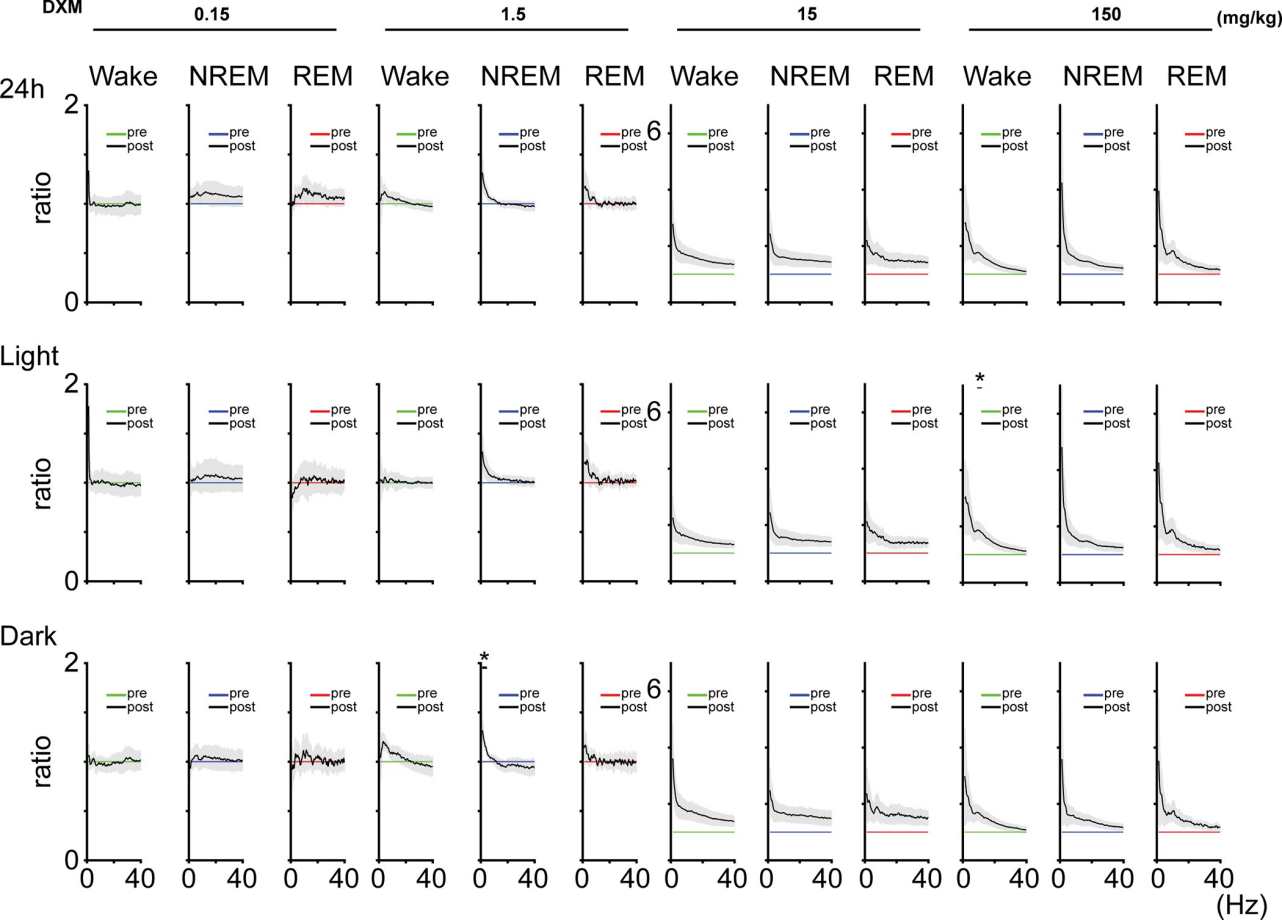
DXM administration increased EEG power. EEG power among each frequency band during the wake (left panel), NREM (middle panel), and REM states (right panel) before and after each dose of DXM administration. The EEG power following DXM administration was normalized to the power before administration. The results of the statistical tests are shown in Table 9 (n = 5 mice in each dose of DXM, total 20 mice; paired *t*-test). ‘pre’ means saline administration (control experiment), ‘post’ means DXM administration.

A previous study showed that acute administration of DXM (12 mg) in ewes caused an increase in fetal EEG power [17]. Therefore, we compared the EEG power between the administration of saline (control) and the administration of DXM. For 0.15 mg/kg DXM administration, the ratio of EEG power before and after DXM administration was generally around 1 across low to high frequencies, indicating that no significant EEG power changes occurred (Fig 5). On the other hand, following 1.5 mg/kg DXM administration, there was an increase in EEG power in the low-frequency range during NREM sleep (delta power became 1.20 ± 0.09 times [mean ± SEM] after DXM administration [daily total]; Light phase, 1.20 ± 0.12; Dark phase,1.21 ± 0.06). In addition, following 15 mg/kg and 150 mg/kg DXM administration caused an increase in EEG power between each frequency band, but most of them did not show significant differences (Fig 5, Table 9). In detail, delta power became 2.20 ± 0.56 times and 2.58 ± 1.06 times, theta power became 1.77 ± 0.35 times and 1.87 ± 0.40 times, sigma power became 1.67 ± 0.28 times and 1.73 ± 0.27 times, beta power became 1.51 ± 0.22 times and 1.37 ± 0.17 times, and gamma power became 1.37 ± 0.16 times and 1.14 ± 0.12 times (mean ± SEM) after 15 and 150 mg/kg DXM administration during wake, respectively (daily total). A similar trend was observed during NREM sleep, delta power became 2.04 ± 0.59 times and 2.72 ± 1.26 times, theta power became 1.64 ± 0.31 times and 1.72 ± 0.39 times, sigma power became 1.60 ± 0.27 times and 1.55 ± 0.25 times, beta power became 1.52 ± 0.24 times and 1.38 ± 0.17 times, and gamma power became 1.45 ± 0.20 times and 1.24 ± 0.12 times (mean ± SEM) after 15 and 150 mg/kg DXM administration, respectively (daily total). Therefore, it can be concluded that DXM may induce changes in EEG power, particularly at high doses, although no clear dose-response relationship could be identified. Furthermore, we examined changes in EEG power per hour after the first four hours of DXM administration similar to the sleep/wake state analysis discussed previously. Following DXM administration, there was a general trend of increased power, but significant differences were observed in only a few segments, and no consistent pattern was observed (Fig 3, Table 4).

**Table 9:** Results of paired t-test for EEG power among each frequency band between before and after DXM administration for the data in Fig 5.

					*P* Value					
		Wake			NREM			REM		
DXM (mg/kg)	EEG	24h	light	dark	24h	light	dark	24h	light	dark
150	gamma	0.29	0.25	0.33	0.13	0.13	0.17	0.22	0.23	0.16
	beta	0.09	0.08	0.11	0.09	0.11	0.10	0.12	0.16	0.11
	sigma	0.05	4.8×10^−2^*	0.07	0.09	0.11	0.09	0.08	0.11	0.10
	theta	0.09	0.08	0.11	0.14	0.15	0.15	0.11	0.13	0.14
	delta	0.21	0.17	0.26	0.24	0.23	0.27	0.24	0.23	0.27
15	gamma	0.08	0.07	0.11	0.09	0.07	0.12	0.10	0.08	0.12
	beta	0.08	0.08	0.10	0.09	0.10	0.09	0.12	0.12	0.12
	sigma	0.07	0.08	0.07	0.09	0.10	0.08	0.12	0.11	0.11
	theta	0.09	0.11	0.09	0.11	0.12	0.10	0.11	0.10	0.12
	delta	0.10	0.14	0.09	0.15	0.15	0.14	0.18	0.16	0.17
1.5	gamma	0.71	1	0.71	0.63	0.83	0.51	0.99	0.77	0.93
	beta	0.74	0.94	0.74	0.91	0.50	0.58	0.97	0.73	0.99
	sigma	0.43	0.78	0.26	0.15	0.29	0.76	0.78	0.62	0.97
	theta	0.25	0.73	0.07	0.14	0.33	0.10	0.45	0.45	0.48
	delta	0.51	0.88	0.30	0.08	0.18	0.02*	0.34	0.29	0.34
0.15	gamma	0.98	0.88	0.86	0.50	0.76	0.91	0.48	0.90	0.98
	beta	0.86	0.90	0.89	0.44	0.70	0.78	0.38	0.84	0.84
	sigma	0.83	0.91	0.75	0.37	0.72	0.71	0.30	0.83	0.64
	theta	0.96	0.94	0.92	0.38	0.74	0.71	0.40	0.96	0.83
	delta	0.64	0.43	0.71	0.35	0.84	0.90	0.67	0.50	0.92

*p < 0.05.

## Discussion

In this study, we examined the relationship between acute DXM administration and changes in sleep architecture in detail and revealed that acute DXM administration causes sleep disturbance, even at very low doses.

Two action mechanisms have been proposed for steroid-related sleep disturbance. First, steroids reduce serum melatonin levels, which are responsible for circadian rhythm formation [10,18]. Second, steroids reduce brain serotonin (5-HT) levels, which are associated with sleep-wake regulation [19,20]. In the former case, an acute administration of DXM (1 mg) to healthy subjects changed melatonin levels for that night [10]. This is consistent with our study in that sleep disturbance occurred immediately after DXM administration. In the latter case, one week of DXM administration in rats caused a decrease in tryptophan hydroxylase (TPH), a late limiting enzyme of 5-HT, in the dorsal raphe [20]. This was a chronic experiment; therefore, it cannot be directly compared to our results, but their results may represent a possible cause.

The present study investigated whether the high-dose DXM administration causes fragmentation of NREM sleep (Fig 2B and 2C), and our results suggest that the wake-promoting neural system may be activated by DXM administration and inhibit NREM sleep. The activity of each neural system under steroid administration is not fully understood, but several pieces of evidence indicate that some neurons and neurotransmitters are affected, and others are not affected by DXM administration. Regarding orexin neurons, which are known as the principal component of the wakefulness-promoting system [10,21,22], a previous study showed that the concentration of orexin protein concentration in the hypothalamus does not change even under DXM administration (5 d at a concentration of 1.5 mg/kg); this indicates that the output of the orexin neuron does not change following DXM administration in mice [23]. Another study shows that intramuscular DXM administration (7 d at a concentration of 1 mg/kg) increased noradrenaline and decreased adrenaline levels and did not alter the serotonin level of the pineal-paraphyseal complex in *Lissemys punctata punctata* [24]. Overall, the previous and present studies provide several insights into the effects of DXM administration on neural activity, but no definitive answer has been obtained. Additional research is needed to fully understand these effects.

In our study, the EEG power in the entire spectrum slightly changed after DXM administration during wakefulness and NREM sleep. A previous human study showed that chronic (5 d) prednisolone administration causes an increase in EEG power in the theta range [25], but the action mechanism is unknown. Friess et al. [26] showed that acute hydrocortisone induction increased the delta and theta power during NREM sleep, and they further showed that it was associated with nocturnal GH release. Another animal model study showed that acute administration of DXM (12 mg) in ewes caused an increase in fetal EEG power [17]. Their study considered histology, but there was no evidence of cell death, tissue injury, ischemic cell change, or activated microglia. Based on these results, it is not surprising that EEG power changes after DXM administration. However, the mechanism of action behind these changes remains unknown, and our study did not demonstrate clear results. Therefore, further experiments are needed to show definitive conclusions.

## Conclusions

Our study demonstrated that acute and very low doses of DXM cause sleep disturbance. These results suggest that sleep disturbance should be addressed even with low-dose DXM administrations in daily clinical practice.
